# Case Report: Combined Intra-Lesional IL-2 and Topical Imiquimod Safely and Effectively Clears Multi-Focal, High Grade Cutaneous Squamous Cell Cancer in a Combined Liver and Kidney Transplant Patient

**DOI:** 10.3389/fimmu.2021.678028

**Published:** 2021-05-27

**Authors:** Dejan Vidovic, Gordon A. Simms, Sylvia Pasternak, Mark Walsh, Kevork Peltekian, John Stein, Lucy K. Helyer, Carman A. Giacomantonio

**Affiliations:** ^1^ Department of Surgery, Faculty of Medicine, Dalhousie University, Halifax Regional Municipality, NS, Canada; ^2^ Faculty of Medicine, Dalhousie University, Halifax Regional Municipality, NS, Canada; ^3^ Department of Pathology and Laboratory Medicine, Faculty of Medicine, Dalhousie University, Halifax Regional Municipality, NS, Canada; ^4^ Department of Medicine, Faculty of Medicine, Dalhousie University, Halifax Regional Municipality, NS, Canada

**Keywords:** intralesional immunotherapy, intralesional IL-2, organ transplant recipient, imiquimod, cutaneous squamous cell carcinoma (cSCC), intralesional, interleukin-2 (IL2), aldara

## Abstract

Cutaneous squamous cell carcinoma (cSCC) is the second most common non-melanoma skin cancer worldwide, with ever increasing incidence and mortality. While most patients can be treated successfully with surgical excision, cryotherapy, or radiation therapy, there exist a subset of patients with aggressive cSCC who lack adequate therapies. Among these patients are solid organ transplant recipients who due to their immunosuppression, develop cSCC at a dramatically increased rate compared to the normal population. The enhanced ability of the tumor to effectively undergo immune escape in these patients leads to more aggressive tumors with a propensity to recur and metastasize. Herein, we present a case of aggressive, multi-focal cSCC in a double organ transplant recipient to frame our discussion and current understanding of the immunobiology of cSCC. We consider factors that contribute to the significantly increased incidence of cSCC in the context of immunosuppression in this patient population. Finally, we briefly review current literature describing experience with localized therapies for cSCC and present a strong argument and rationale for consideration of an IL-2 based intra-lesional treatment strategy for cSCC, particularly in this immunosuppressed patient population.

## Introduction

Cutaneous squamous cell carcinoma (cSCC) is the second most common non-melanoma skin cancer worldwide (1, 2), and its incidence is steadily increasing yearly (3). While the mortality rates for almost all other forms of cancer decline, the age-standardized mortality rate of cSCC continues to rise. Despite being vastly outnumbered in incidence by basal cell carcinoma (BCC) (4), cSCC is associated with a significantly higher mortality (5). Indeed, in the United States alone, mortality figures for cSCC are now comparable to those of melanoma; a less common disease which is far more lethal (5, 6). Of the nearly 1 million new cases of cSCC each year, there are an estimated 15,000 deaths, compared to 9730 in the case of melanoma (7, 8).

Initial treatment strategies for cSCC include electrodessication and curettage, surgical excision, cryotherapy, or radiation treatment (9). Surgical excision is considered the standard treatment of cSCC, and is able to cure 90% of cSCC cases with a 5-year recurrence rate of 8% and 5-year metastasis rate of 5% (9). Surgery, however, is not always possible, as on occasion, a cSCC is unresectable or is confined to cosmetically or functionally sensitive area. In addition to inoperable cSCCs, a small percentage of cSCCs are aggressive and refractory to standard dermatologic therapies (5, 10, 11). This subset of cSCC, known as aggressive (or high-risk) SCC, has a substantially higher rate of metastasis and associated morbidity and mortality (12, 13). Features consistent with aggressive cSCC include tumor size ≥ 2cm, evidence of perineural invasion, bone invasion or erosion and invasion beyond subcutaneous fat (14). It is noteworthy that high grade histology is also still considered a feature of aggressive disease in the BWH cSCC classification system but no longer in the AJCC 8^th^ edition of cSCC classification (15). High risk cSCC in the context of a history of local recurrence or immunosuppression predicts a significantly higher risk of disease recurrence, metastasis and disease specific mortality.

Herein, we describe the novel utilization of two immunomodulatory local therapies, intralesional IL-2 and topical 5% imiquimod, used in conjunction to achieve complete remission in a double solid order transplant recipient with no evidence of inducing immune-mediated organ rejection.

### Case Description

#### A Case of High Grade, Multi Focal, Rapidly Progressing Cutaneous Squamous Cell Carcinoma in a Double Solid Organ Transplant Recipient

**Figure 1:**
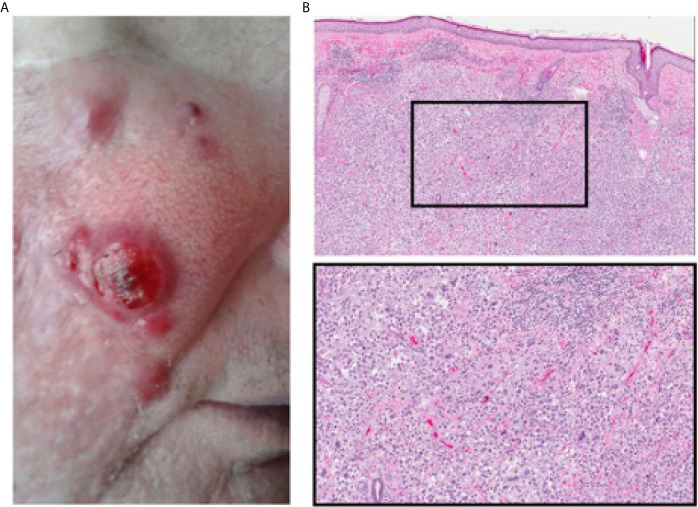
Pre-treatment recurrence of facial cSCC. **(A)** Extent of disease on the right cheek prior to starting treatment with intra-lesional IL-2. **(B)** Histological profile of facial lesions prior to starting intra-lesional IL2 (top 40X, bottom 100X magnification), showing poorly differentiated cSCC.

**Figure 2:**
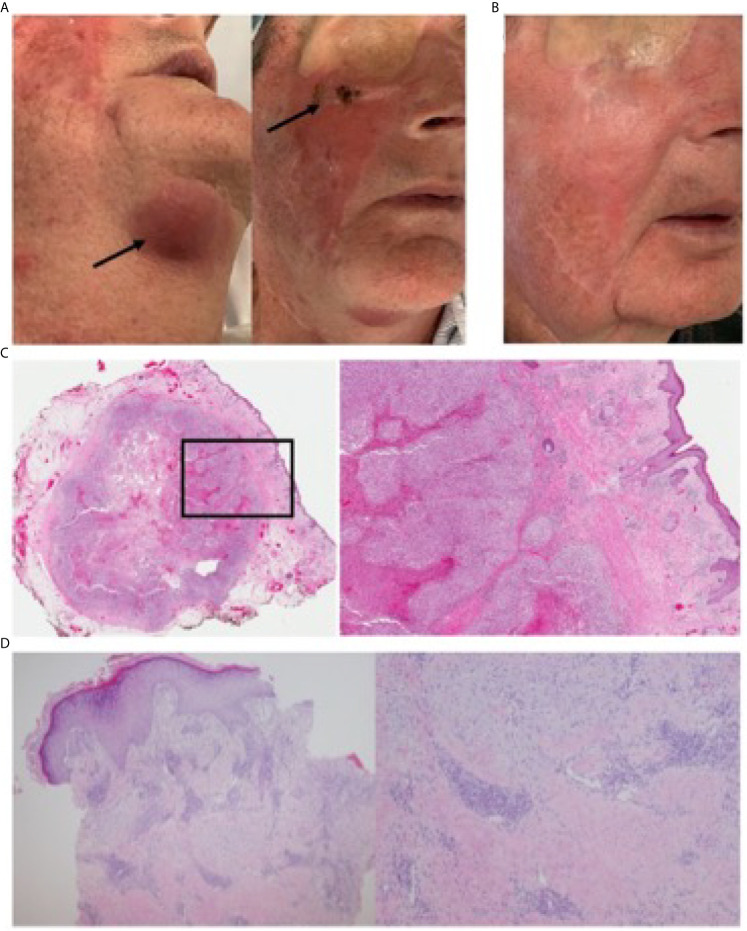
Post-treatment course with intra-lesional IL2 and imiquimod. **(A)** Complete response of facial lesions with resolving submandibular nodule. Arrows identify nodule and infraorbital area of healing (biopsied) skin. **(B)** Sustained complete resolution of facial cSCC three months following completion of treatment. **(C)** (left) Histological profile of excised submandibular nodule low magnification; (right) 20X magnification reveals residual high-grade cSCC with necrosis, a pronounced lymphocytic infiltrate, with no evidence of nodal tissue **(D)** (left) 40X magnification of biopsied, healing infraorbital skin showing ulceration and complete clearance of cSCC; (right) 100X magnification.

Our patient, a 72-year-old male with a past medical history of polycystic kidney disease, received a liver and kidney transplant from a single donor in January 2006; however, this first kidney immediately failed. He subsequently received a second living-related (i.e. from a living family member) donor kidney in January 2008. His initial anti-rejection medications included tacrolimus (4 mg daily), mycophenolate (2 g daily) and prednisone (10 mg daily). From a transplantation perspective, he tolerated this regimen well. In 2009, however, he developed a small area of cSCC just above his right eyebrow. This was locally excised with clear margins. Cutaneous squamous cell cancer recurred at the same site over the right eye in 2014 and again in 2015. His second recurrence was characterized as well-differentiated cSCC, resected with clear margins but associated with perineural invasion. He received targeted radiation of 5000cg to the surgical site. In Aug 2017, cSCC recurred in the skin along the supraorbital rim, again with perineural invasion. Due to the extent of this recurrence, he received a radical excision with right eye enucleation and radial forearm skin graft to repair the resulting facial skin defect. All margins were reported negative. By this point, the patient’s immunosuppression medications had been modified and included sirolimus (1 mg oral daily), tacrolimus (1 mg oral daily), and prednisone (5 mg oral daily). Following his surgery, the tacrolimus dose was reduced (to 0.5mg every two days), and the sirolimus dose increased (to 2 mg oral daily) in hopes of reducing the risk of developing further cSCC. However, he went on to develop a small cSCC on the right side of the nose; treated with excision and local radiation. He subsequently developed two new lesions in April and May of 2019, both excised with narrow margins. In January 2020 he developed a further two new lesions, one in the skin overlying the right zygoma and a second at the margin of the radial forearm skin graft on the right cheek. Margins were now involved. The area was treated with targeted radiation, taking care to avoid significant radiation field overlap from previous treatments. In February 2020 five new lesions were identified (**Figure 1A**). Pathology now demonstrated poorly differentiated cSCC with lymphovascular invasion and positive margins (**Figure 1B**). At least one of the lesions was felt to be metastatic. Consultation was made for consideration of systemic immunotherapy; however, being a liver and kidney transplant recipient, it was felt that systemic immunosuppression would confer significant risk to failure of both transplants. Intra-lesional immunotherapy was offered as a potentially safer experimental alternative. After careful consideration of the options and associated potential risks and benefits, weekly injections of intra-lesional IL-2 (8M IU total dose per session, divided among multiple lesions and sites of injection) was initiated. Initially, he experienced partial regression in some lesions but clinical progression in others, with developmental of a new submandibular nodule deep to the skin. This subcutaneous nodule was treated intra-lesional injections of IL-2 (bringing the total dose administered to 10M IU per session). Additionally, at this time imiquimod (a topical TLR-7 agonist) was added to all facial lesions, administered as a thin film once daily for five out of seven days per week, by the patient. This was done in the hopes of augmenting an IL-2-mediated anti-tumor immune response. Over the course of the following six weeks, all facial lesions completely clinically responded. In addition, the subcutaneous, submandibular nodule had significantly diminished in size (**Figure 2A**). For a number of reasons, including patient-reported severe pain with injections particularly at the site of the submandibular lesion, ongoing cost of medications, and costs associated with travelling back and forth for weekly treatment, we elected to proceed with excision of the residual submandibular lesion; at the same time, a representative biopsy of the right facial skin was taken as well. The submandibular lesion demonstrated residual high-grade cSCC with necrosis and a pronounced lymphocytic infiltrate but no evidence of nodal tissue (**Figure 2C**). Histological analysis of the right cheek skin revealed no evidence of any residual cSCC (**Figure 2D**). Margins were clear and no lymphovascular invasion or perineural invasion was identified. The area remains disease free 3 months post-treatment (**Figure 2B**). During the course of treatment, liver and kidney function was closely monitored and unaffected by the localized treatment strategy. Overall, he experienced no decline in either (kidney or liver) graft function and had no signs of rejection.

## Discussion

### The Immunopathological Basis of cSCC in Solid Organ Transplant Recipients

The immune system plays a vital role in the pathogenesis and progression of cSCC (16). Indeed, the major risk factors for cSCC development include genetically defined skin type, chronic UV exposure, chronic skin damage, and immunosuppression (17–21). The impact of immunosuppression on cSCC development has been studied most thoroughly in the context of solid organ transplant recipients. While immunosuppression is necessary to prevent transplant rejections, lifelong use of these agents has been shown to promote carcinogenesis, with cSCC being one of the most common in these patients (22).

The prominence of cSCC development in patients with iatrogenic immunosuppression strongly suggests that cSCC may have an inherent ability – that other cancers lack – to circumvent cancer immune surveillance. It has been shown in a large series of renal transplant patients that immunosuppression increases cSCC formation up to 250-fold in comparison to immunocompetent patients (23–25). The degree of immunosuppression may correspond to cSCC incidence, where reduction of immunosuppression reduces the total number, and rate of formation of cSCC (26). Numerous immunosuppressive drugs have been linked to cSCC development, namely calcineurin inhibitors (26, 27), glucocorticoids (28–30), and biologics (infliximab (31, 32), etanercept (32–34), adalimumab (32)). Furthermore, when solid organ transplant recipients do develop cSCC, their tumors tend to be more aggressive and carry a higher risk for metastasis (35). Indeed, iatrogenic immunosuppression *via* calcineurin inhibitors inhibit Langerhan’s cells (36, 37), dermal dendritic cells (38, 39), and T-cell signaling and proliferation, and cyclosporine directly promotes tumor development (40–42). Calcineurin inhibitors effectively disrupt IL-2 production, and as such are able to dampen immune response to allogenic antigens – a desired effect when trying to persevere tolerance towards solid organ transplants; however, this iatrogenic immunosuppression comes at a price, and significantly impairs cancer immunosurveillance (23–27). Unlike calcineurin inhibitors, the mammalian target of rapamycin (mTOR) inhibitors block IL-2 induced signal transduction, and does not abrogate IL-2 production completely, thereby allowing some functions of IL-2 to remain intact (43). As such, recently mTOR inhibitors, which include sirolimus (rapamycin), temsirolimus, and everolimus, have garnered favour because they have a lower association with *de novo* skin malignancies, and may in-and-of themselves have a direct anti-tumor effect. In retrospective analyses renal transplant recipients who received either sirolimus or everolimus without cyclosporine had a reduced number of *de novo* skin malignancies (44), and some patients experienced regression of skin cancers such as Kaposi’s sarcoma (KS) and SCC that were present prior to initiation of mTOR therapy (45–49). For these reasons, in our high-risk patient presented above, recurrent cSCC was the major factor in deciding to switch him from tacrolimus to sirolimus early on. However, he unfortunately continued to present with recurrent cSCC.

The substantially higher incidence of cSCC in immunosuppressed patients underscores the impact of the immune system in cSCC susceptibility and pathogenesis. Indeed, variations in immunological makeup may influence the ability of human hosts to recruit adaptive immune responses needed to prevent cSCC development (50). Class I and class II HLA genes encode major histocompatibility complex (MHC) proteins which allow for presentation of antigenic peptides such as tumor antigens to CD8+ and CD4+ T-cell lymphocytes, respectively. Variations in MHC proteins have been implicated in multiple cancers by influencing host defenses against tumorigenesis (50). Aberrant expression of both class I and class II HLA proteins on the surface of cSCC cancer cells is reported in both immunocompetent and immunosuppressed patients (51–55). Abnormalities in class I and class II HLA proteins are well documented in cSCC cells, reinforcing the notion that cSCC pathogenesis is inherently connected to faulty immune regulation. Several clinical studies have indicated that specific class I HLA germlines may predispose the development of cSCC in immunosuppressed patients (56–58). It has also been proposed that aberrant expression of class II HLA proteins on cSCC cancer cells may facilitate tumor escape from host defense mechanisms, as seen in other cancers including HNSCC and acute myeloid leukemia (59, 60). Furthermore, cSCC has the ability to downregulate the presentation of highly immunogenic neoantigens to TCRs (61). Thus, an important facet in the mechanism of cSCC immune escape is HLA and neoantigen dysregulation; targeting mechanisms that improve tumor-associated antigen presentation may thus be useful in the immunotherapy of cSCC.

Another avenue through which cSCC mediates immune escape is through local cytokine dysregulation. For example, cSCC significantly downregulates CCL27, a chemokine that promotes T cell homing to skin, throughout its progression from AK to malignant cSCC (62). cSCC tumors that tend to be deeper and more advanced also significantly upregulate CXCR7, which signals through CXCL12 to promote ERK signaling, thus prolonging tumor cell survival (63). Cytokine profiling of tumors reveals that in the progression to malignant cSCC, precancerous lesions dramatically upregulate production of IL-6 (64), a proinflammatory cytokine that has previously been shown to augment cSCC growth through modulation of pro-tumorigenic cytokines and angiogenic factors (65). Therefore, therapies that modulate the local cytokine and chemokine profile of cSCC may be of benefit.

### Systemic Treatment of cSCC

Aside from surgical methods, there is a paucity of treatment modalities for aggressive cSCC. Systemic therapies for cSCC have shown limited success, although rigorous assessment of systemic therapies has been limited (66). To date, a number of systemic therapies have been used to treat cSCC, including: chemotherapeutics (cisplatin (67–69), 5-fluorouracil [5-FU] (67, 68, 70, 71), bleomycin (67), and doxorubicin (69)), 13-cis-retinoic acid (13cRA (72)), immunotherapies (interferon-α2a [IFN-α] (72)), gefitinib (73) and cetuximab (74) (agents targeting epidermal growth factor [EGFR]), and more recently nivolumab (75) and cemiplimab (76) (PD-1 immune checkpoint inhibitors).

Although chemotherapeutics have enjoyed modest success in treating surgically unresectable, and metastatic cSCC, they are accompanied by a wide range of – sometimes intolerable – gastrointestinal, hematologic, and metabolic side effects (67–69). Studies using 13cRA and IFN-α to treat SCC are conflicting; in the only trial using these compounds as adjuvant therapies in cSCC, they were ineffective (66, 72). In recent years targeting EGFR has shown some promise; indeed, EGFR is implicated in a variety of cancers including non-small cell lung cancer, colorectal cancer, pancreatic cancer, and head and neck squamous cell carcinoma (HNSCC) (77–81). Insofar as treating cSCC, two candidates have recently made it through phase II clinical trials: cetuximab, a humanized monoclonal antibody targeting EGFR, and gefitinib, which inhibits ATP-binding to EGFR (73, 74). Both compounds showed modest complete response rates, albeit with moderate toxicity.

The moderate to severe toxicities of systemic agents used to treat aggressive cSCC makes it challenging to use these drugs in the context of high-risk patients with significant comorbidities and chronic conditions, such as SOTRs. Additionally, the use of any systemic immune modifying agents may trigger immune-related adverse events (irAEs) (82) that could manifest as life-threatening (i.e. organ rejection) in patients who are iatrogenically immunosuppressed; thus, they are generally not recommended for use in this clinical context (83). Therefore, it is imperative that therapies that minimize systemic toxicities while maximizing the local tumor clearance be studied further.

### Intra-Lesional and Topical Therapies

The serious toxicity profile associated with systemic therapies may be altogether avoided by treating instead with intra-lesional injections. Prior studies have revealed lowered rates of toxicity associated with intra-lesional injections when compared with systemic administration. Furthermore, by delivering an increased concentration of the active agent at the site of action, increased rates of efficacy are observed (84, 85). To date, only a handful of intra-lesional agents have been used for treatment of cSCC, although this method of delivery has been extensively studied in melanoma wherein systemic adverse events are minimized and the local immune response is maximized (86).

Several case reports and small trials have been reported where actinic keratosis (AK) has been treated successfully using intra-lesional therapies (87, 88). Furthermore, 5-FU (70), methotrexate (MTX) (89), several INFs (90–93), and bleomycin (94) have all shown some utility in treating both AK and cSCC, although the data for cSCC is somewhat limited. The vast majority of reports of intra-lesional treatments of cSCC’s are case reports; however, most show good response rates and limited side effects, with the majority of side effects reported including erythema, pain and swelling at the site of injection, and occasionally, mild fever and chills. Indeed, when reflecting on our patient presented above, he experienced no immune-related adverse events. Remarkably, in a patient who cannot tolerate T cell activation (due to risk of graft failure), local injection of the potent T cell activator IL-2 was sufficient to maximize the anti-tumor immune response and did not cause any further toxicities. The patients’ side effects were limited to pain and swelling at the injection site with a short period of chills following injection, further demonstrating the benefit of intra-lesional immune therapies compared to systemic therapies.

Topical therapies that are applied locally can also mitigate the risk of systemic adverse events. A number have achieved moderate success in the treatment of AK, such as topical 5-FU, imiquimod, ingenol mebutate, and diclofenac, which are all FDA approved for this indication (95). With regards to cSCC, topical 5-FU is also commonly used (96). Additionally, early randomized controlled trials show a high degree of clinical benefit from topical imiquimod in the context of cSCC, with approximately a 70% complete response rate (97, 98). Of particular importance, imiquimod is a potent Toll-like receptor 7 (TLR7) agonist that induces local cytokine changes to cause a shift in the immunological balance intratumorally (99).

These therapies individually or in combination therefore represent a possible treatment modality in the context of high risk cSCC. In solid organ transplant recipient patients who cannot tolerate other therapies, or have failed standard local treatment with surgery and/or radiation, use of intra-lesional and/or local therapies may provide substantial clinical benefit. As iatrogenic immunosuppression plays a role in mediating immune escape of these patients’ tumors, identifying local therapies that can counteract that process is paramount.

### Augmenting the Anti-Tumor Immune Response in cSCC to Prevent Immune Escape

As briefly discussed above, one of the avenues through which cSCC mediates immune escape is by downregulation of cytotoxic T cells. Thus, local therapies that promote T cell proliferation and activity are of particular interest. Outside of case reports, there are very few studies examining the immunological response to intra-lesional therapies in cSCC. Neoadjuvant intra-lesional MTX is able to induce lymphocytic inflammatory infiltrate, although the cell types and their role within the infiltrate are unclear (100). IFNα-2β;, another immunological treatment that was used intra-lesionally in a number of early studies achieved moderate clinical success, with a 88.2% complete response rate, but the specific mechanism of action is still unclear (101). However, extrapolating from its function in other contexts, it is likely upregulating a T cell-mediated anti-tumor response through the JAK1/STAT1 pathway (102). Unfortunately, in the years since these early studies, IFNα-2β; has fallen out of clinical investigation.

Interestingly, other potent T cell activating immunotherapies such as IL-2, have not yet been studied in the context of cSCC, to our knowledge. This is despite its extensive investigation as an intra-lesional agent in melanoma (86, 103–105) and HNSCC (106–109), where it mediates a shift to CD8+ T cell-mediated tumor clearance. The excellent response demonstrated by this case warrants further investigation into the possible role intra-lesional IL-2 may have in the treatment of cSCC.

Imiquimod, the other local agent used in this case, applied topically to cSCC is able to induce numerous changes targeted at augmenting T cell effector function. First, imiquimod causes dense CD8+ T cell infiltration into treated tumors, which produce significantly higher amounts of IFNγ, perforin, and granzyme compared to untreated tumors (99). In addition, treatment with imiquimod causes a shift to a polarized Th1 cytokine response (110). Production of IL-10 and TGF-β, which are known cytokines responsible for cSCC immune evasion (111), were significantly downregulated following imiquimod treatment. Imiquimod also antagonizes cSCC-mediated vascular remodeling, by upregulating E-selectin to promote cytotoxic T cell homing to the tumor (112). Furthermore, it significantly decreases FOXP3+ Treg cell levels in treated tumors, and also inhibits their function (112). Most importantly, imiquimod-treated tumors exhibit clonally expanded CD8+ T cell repertoires (112), suggesting a specific anti-tumor immune response and possibly adaptive immunity.

## Conclusion

The case presented herein provides an excellent example of the complexity of the pathobiology and clinical behavior of cSCC in the immunosuppressed patient population. The primary objective of immunosuppressive regimens in solid organ transplantation is prevention of acute allograft rejection through IL-2 blockade. Calcineurin inhibitors such as tacrolimus prevent IL-2 production while sirolimus inhibits IL-2 receptor signal transduction *via* action on mTOR. These complementary mechanisms of action align to effectively preventing acute allograft rejection while at the same time compromising both innate and adaptive immune pathways.

Carcinogenesis in cSCC may be associated with a number of cellular modifications that facilitate immune escape including aberrant HLA expression, downregulation of important chemokines associated with T-cell homing and production of other cytokines such as IL-6, a cytokine having a myriad of functions including promoting angiogenesis, tumor cell growth and an overall proinflammatory and pro-tumorigenic response. In the setting of an immunocompromised host, cSCC is therefore ‘facilitated’ to evade the body’s natural cancer immune surveillance mechanisms *via* the aforementioned mechanisms. Our patient began experiencing cSCC within a year of his second kidney transplant. His initial lesions were small, well differentiated cSCC’s. However, early on in his disease he developed a high-risk feature, that being perineural invasion. Despite achieving clear surgical margins and receiving adjuvant radiation to the field, the disease recurred. Over time, the recurrences became more frequent, with increasing numbers of high-grade features including lesions greater than 2cm, perineural invasion and eventually transformation to high grade histology. At this point, further surgery was deemed futile and with no further options for radiation an alternate treatment strategy needed to be considered.

With recent randomized evidence to support systemic immunotherapy in cSCC, the option of systemic treatment with a PD-1 inhibitor was initially discussed. Given that there is currently a paucity of evidence to support safe delivery of systemic immune therapy in the solid organ transplantation population, and with both a liver and a kidney allograft at risk of rejection, we decided against systemic immunotherapy. However, we have developed local experience and success with IL-2 based intra-lesional treatment of cSCC in some of our kidney transplant patients. Our rationale for an IL-2 based treatment strategy in this population is based on the knowledge that current immunosuppressive regimens target IL-2 production or its effects systemically. Our hypothesis is that local re-introduction of IL-2 into skin bearing cSCC and specifically into the tumor microenvironment will serve to restore both the innate immunity, and effector T-cell function lost through iatrogenic IL-2 suppression thereby re-establishing effective cytotoxic immunity against cSCC. The initial observed response to IL-2 monotherapy was mixed with some lesions regressing while others progressed. We were concerned that significantly increasing the local IL-2 dose beyond the 10M IU (total injected dose) might systemically impact immunity and potentially initiate allograft rejection. Therefore, we added topical imiquimod with the intention of potentiating the local cytokine response promoting T-cell and NK-cell homing and effector function. The combination proved highly effective with clearance of all facial lesions and marked regression of the subcutaneous, submandibular nodule making surgical excision much easier to achieve with a clear margin. To help reduce the development of further cSCC’s, the patient’s calcineurin inhibitor dose was further reduced.

The foundational concepts of cSCC tumorigenesis in the immunocompromised population are aberrant HLA expression, alteration of chemokine and cytokine profiles, and T cell dysfunction, as discussed prior. In our patient, we used two immunomodulatory agents in conjunction in an attempt to modulate some of the aforementioned pathways in the favor of an anti-tumor response, while mitigating systemic immune toxicity. We acknowledge that it is not fully clear how IL-2 and imiquimod may act in conjunction to mediate these anti-tumor immune responses; this merits further mechanistic study.

Herein we demonstrate, for the first time to our knowledge, that an IL-2 based intra-lesional treatment strategy safely and effectively treated multiple high grade cSCC lesions in an immunocompromised, multi-organ transplant patient. It is arguable that given the biological propensity for cSCC to evade immune systems in the setting of iatrogenic immunosuppression, an IL-2 based intra-lesional immunotherapy treatment strategy should be first line consideration for all cSCC lesions. Furthermore, from a patients’ perspective, offering patients a minimally invasive, localized therapy gives them the opportunity to forego complex surgical management, which in some cases can be undesirable cosmetically or may carry major perioperative risks in this population.

## Data Availability Statement

The original contributions presented in the study are included in the article/supplementary material. Further inquiries can be directed to the corresponding author.

## Ethics Statement

Ethical review and approval was not required for the study on human participants in accordance with the local legislation and institutional requirements. The patients/participants provided their written informed consent to participate in this study.

## Author Contributions

DV and CG contributed to conception and design of this manuscript, and wrote sections of the manuscript. GS wrote sections of the manuscript. SP provided data for figures within the manuscript. MW, KP, SJ, and LH contributed to project conception and design. All authors contributed to the article and approved the submitted version.

## Conflict of Interest

The authors declare that the research was conducted in the absence of any commercial or financial relationships that could be construed as a potential conflict of interest.
